# Waiting times in emergency departments: a resource allocation or an efficiency issue?

**DOI:** 10.1186/s12913-020-05417-w

**Published:** 2020-06-17

**Authors:** Milena Vainieri, Cinzia Panero, Lucrezia Coletta

**Affiliations:** 1 Associate Professor at Management and Health Laboratory, Institute of Management, Scuola Superiore Sant’Anna, Pisa, Italy; 2 Post-doctoral researcher at Università degli studi di Genova, Genoa, Italy; 3 PhD candidate, Management and Health Laboratory, Institute of Management, Scuola Superiore Sant’Anna, Pisa, Italy

**Keywords:** Emergency departments, Waiting times, ED admission rates, Overcrowding, Efficiency, Resource allocation

## Abstract

**Background:**

In recent years, the flow of patients to the Emergency Departments (ED) of Western countries has steadily increased, thus generating overcrowding and extended waiting times. Scholars have identified four main causes for this phenomenon, related to: continuity of primary care services; availability of specific clinical pathways for chronic patients; ED’s personnel endowment; organization of the ED. This study aims at providing a logical diagnostic framework to support managers in investigating specific solutions to be applied to their EDs to cope with high ED waiting times. The framework is based on the ED waiting times and ED admission rate matrix. It was applied to the Tuscan EDs as illustrative example.

**Methods:**

To provide the factors to be analyzed once the EDs are positioned into the matrix, a list of issues has been identified. The matrix was applied to Tuscan EDs. Data were collected from the Tuscan performance evaluation system, integrated with specific data on Tuscan EDs’ personnel. The Tuscan EDs matrix, the descriptive statistics for each quadrant and the Spearman’s rank correlation analysis among waiting times, admission rates and a set of performance indicators were conducted to help managers to read the phenomena that they need to investigate.

**Results:**

The combined reading of the correlations and waiting times-admission rates matrix shows that there are no optimal rules for all the EDs in managing admission rates and waiting times, but solutions have to be found considering mixed and personalized strategies.

**Conclusions:**

The waiting times-admission rates matrix provides a tool able to support managers in detecting the problems related to the management of ED services. In particular, using this matrix, healthcare managers could be facilitated in the identification of possible solutions for their specific situation.

## Background

In recent years, in many Western countries the flow of patients to the Emergency Departments (ED) has constantly increased [1]. This flow has often determined the ED overcrowding [2–6], that occurs every time the number of the waiting patients exceeds the available resources, in terms of beds and/or personnel. Therefore, overcrowding is a phenomenon that seriously limits the hospital functions [7] in terms of both delays in the patients’ care and poorer outcomes [8–11]. Overcrowding is also associated to the dissatisfaction of both physicians and nurses, working under pressure, and patients waiting to be treated [12]. In particular, the waiting times are among the most important causes of ED patient dissatisfaction [8] and they negatively influence patients’ perception of the service quality [13]. Complaints of dissatisfied patients are often vividly reported on the media thus causing pressure on policy makers and hospital managers.

This trend seems irreversible, because it is based on the evolution of health expectations and needs of the populations. This led a high number of scholars focusing on the factors affecting the overcrowding and ED waiting time.

In particular, these factors can be grouped into four main categories: 1) how primary care and continuity are organized; 2) the existence and effectiveness of organizational models and clinical pathways for chronic patients; 3) the presence of bottlenecks related to ED’s personnel or equipment endowment; 4) how the ED is organized and its connection with the rest of the hospital. The first two are related to the admissions to the ED services, the others to the way the ED and the hospital are organized to manage the flow of the patients.

With reference to the first group of factors, how primary care and continuity are organized, some authors underlined that overcrowding may depend on the high number of non-urgent patients seeking help from the ED [14, 15], while these patients could turn to other health settings, namely primary care. There could be several reasons behind this patient’s choice such as the capacity of ED to provide a full, timely service, including diagnosis and examinations [16, 17] as well as the higher perceived quality of ED services [18]. However, there are scholars outlining that admissions to the EDs are higher when there are problems related to the supply side, in particular when primary care services fail to respond to patients’ needs [17, 19–25].

The second group of factors influencing the ED admissions is related to the existence and effectiveness of organizational models and clinical pathways for chronic patients. Indeed, since the beginning of 2000 disease management programs have been proposed, like the chronic care management [26, 27] with the aim to improve the health conditions of the patients [28], by identifying the health needs before the disease appears or it becomes serious. These programs may be particularly relevant to cope with potential avoidable ED access, considering that chronic patients are frequent users (i.e. patients with at least four ED admissions per year) [29–33].

A third group of factors influencing the waiting times in ED is related to potential bottlenecks such as the ED structural endowment of physicians and nurses (and rooms). Some studies outlined that the waiting times may depend on the insufficient number of physicians and nurses [34–36] or equipment like the existence of diagnostic imaging reserved to the ED [37, 38].

The last group of factors influencing the ED overcrowding and the waiting times concerns how the ED is organized and its integration with the rest of the hospital. For instance, the existence of fast track for specific health problems (such as pregnancy or eye issues) may reduce waiting times because patients going to ED for these conditions have been taken in care directly by the specialists’ ambulatories or wards thus helping the ED personnel to cope with the visits’ demand [37, 38].

Another aspect related to the organization is the boarding, that occurs when patients needing a hospitalization have to wait in the ED because the ward beds are not available [3, 35, 39, 40]. This implies that the effort of ED personnel is diverted, at least in part, from the new patients that come to the ED because they have to pay attention also to patients waiting for being hospitalized [41].

To support hospital managers to cope with the third and the fourth groups of factors affecting the ED waiting times, some scholars have proposed operation or lean management approaches [42, 43]. Whilst to cope with the first two groups of factors, many scholars suggested to re-arrange primary care or healthcare pathways. However, how to discover which is or are the factors that may affect the waiting times in a specific ED is an issue still uncovered in literature. It is a topic often left in the hands of hospital managers who have to analyze their own data. It may result also difficult because of the possible bias coming from the lack of comparisons (such as the definition of personnel endowment) or the lack of information at hospital level (such as the primary care efficiency or the effectiveness of the healthcare pathways).

This study aims at providing a logical framework that both the meso-level of government (such as Regional governments) and hospital managers can use as a logical diagnostic tool to understand their specific positioning with reference to the different potential factors influencing the ED waiting times and, therefore, to support them to find the solutions that can suit their EDs. This diagnostic logical framework was applied to the Tuscan health system to illustrate how to read it.

## Methodology

### Designing the logical framework to investigate ED waiting times

To provide a diagnostic logical framework to detect the specific situation and the factors that potentially influence ED waiting times, we propose a descriptive study, based on a matrix that compares the ED waiting times with the ED admission rate. This framework has been designed and applied to other services [44, 45]. It is based on a matrix that compare performance service waiting times and service use-rates. The position into the matrix allows to rapidly realize if the waiting times or the service use rates are higher or lower than the median of the other units observed in a specific geographical area. The four quadrants coming from the use of median value for waiting times and service use-rates identify four situations (higher waiting times higher service use rates; lower waiting times lower service use rates; higher waiting times lower service use rates; lower waiting times higher service use rates) that may require different strategies. Strategies need to be personalized on the basis of the service analyzed. Hence, in order to adapt this matrix to the ED services we followed three steps.

The first step was to select indicators that can be monitored to detect the factors associated with the ED waiting times and ED admission rates. Indicators were based on both the categories identified in the literature, reported in the first paragraph, and the consolidated experience of the performance evaluation system in the Tuscany Region [13], which is using more than 300 performance indicators also covering ED and primary care services also share with other Italian Regions [36, 46–48]. This experience guarantees that the indicators’ selection already received a validation process by professionals and healthcare managers [36, 47].

The second step was to propose for each quadrant the issues to be investigated in order to cope with ED waiting times and ED admission rates. Hence, we identified the indicators that we suggest to be analyzed for each quadrant to disentangle why the ED got that performance in terms of waiting times and admission rates.

The third step was to apply this logical framework to the real world data presenting it to ED heads of departments in the Tuscany Region. The illustrative example was coupled with the correlation analysis of the factors identified in the first step.

### Study setting

This matrix was applied to the Tuscan EDs data to better highlight the support that this diagnostic logical framework can provide to managers and policymakers in coping with EDs’ waiting times.

Tuscany is a medium-size Italian Region, with a population of 3,75 million with a good level of performance of its healthcare services [46]. However, there is a wide variability of performance results among its Districts and EDs. Consistently with the international trend, the number of admissions to the Tuscan EDs increased by 5,4% in the last 7 years arriving at 1 million and half of admissions.

The service is provided by 38 EDs, which refer to 3 LHAs and 4 Teaching Hospitals, and are grouped into 25 territorial Districts.

In 2018, the overall admission rate to the EDs per 1.000 inhabitants was 361.49, with a great variability among the Districts (from 279.99 to a maximum of 556.49) and among the ED admission rate referred to minor priority codes, that which was 88.28 per 1.000 inhabitants (from 43.59 to a maximum of 146.52). With reference to the waiting times, the median waiting time is 72 min, from a minimum of 36 min to a maximum of 282 min. Urgent priority codes, which need immediate admission to the treatment, are included.

### Data analysis

The analysis conducted is a qualitative description of the application of the diagnostic logical framework to the Tuscany data.

The data concerning the ED admission rates, the waiting times and the performance of primary care were retrieved from the publicly disclosed data on Tuscan performance evaluation system (https://performance.santannapisa.it/); data related to personnel come from a research report [36]. For what concerns the data about the endowment of ED personnel, we used the last data available (2015), whereas all the other data are updated to 2018.

To design the matrix, we linked ED admission rates, computed at the District level, and ED waiting times, computed at the hospital level. Therefore, as a methodological criterion, for those Districts which comprehend more than one hospital, we considered the waiting times of the prevalent ED. The number of admissions of the selected EDs represents always more than 70% of the admission per inhabitants. The reference lines that identify the quadrants represent the regional median values. We reported some descriptive statistics for the four quadrants to illustrate how this matrix can help managers to detect their situation.

We reported some descriptive statistics for the four quadrants to illustrate how this matrix can help managers to detect their situation.

To complete the study a correlation analysis among variables was performed with a level of significance at 10%. We executed the Spearman’s rank correlation because most of the variables were not normally distributed. The correlation analysis may help to identify common patterns among the Tuscan EDs in association to the factors analyzed, suggesting that some issues may require a regional intervention.

## Results

### The diagnostic logical framework to cope with ED waiting times

The selected indicators where presented in Table 1. In particular, for factors related to primary care and continuity (group 1), we investigated the GP’s density; for factors related to the existence and effectiveness of chronic management programs (group 2), we considered the indicators of the avoidable hospitalizations as well as the enrolment into the Tuscan chronic care program monitored by the Tuscan performance evaluation system [48]; for factors related to the presence of bottleneck (group 3) we used the indicators coming from a Tuscan research on EDs’ personnel [36]; for factors related to EDs performance and hospitals’ organization (group 4) we considered all the indicators referred to the Tuscan performance evaluation system. These indicators cover both quality aspects (such as the number of EDs readmission) and the appropriateness (such as the percentage of hospitalized patients admitted to the ward within 8 h) [48].

**Table 1:** Variables taken into account

**Groups**	**Variables**
ED admissions	ED admission rate per 1000 inhabitants
ED waiting times	ED waiting times
Group 1: Continuity of care	Number of GPs per 1000 inhabitants in the district
Group 2: Chronic care	Percentage of inhabitants (≥ 16 years) enrolled in Tuscan chronic care programmes
	Primary care effectiveness of chronic disease management (hospitalization rate for heart failure, diabetes, chronic obstructive pulmonary disease)
Group 3: ED personnel endowment	Number of FTE ED physicians per 10,000 admissions
Group 4: Organizational factors	% of non-urgent treated within four hours
	% admissions to the observation unit
	% of hospitalized patients from the observation unit
	Observation unit length of stay < 6 h
	Observation unit length of stay > 48 h
	% admissions to the observation unit
	% of hospitalized ED patients
	% hospitalized ED patients within eight hours
	Bed occupancy rate
	% appropriateness of surgical setting
	% of patients hospitalized in the intensive care unit within 24 h
	% of repeat admissions to the ED within 72 h
	% Patients left without being seen

**Fig. 1:**
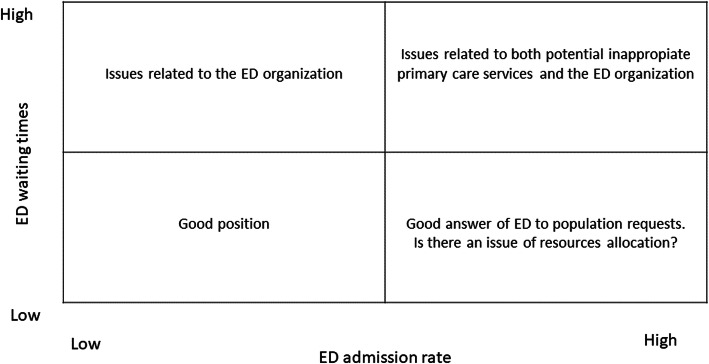
A scheme for the waiting times-admission rate matrix

Positioning the EDs inside the four quadrants allows to draw down a list of potential factors affecting that performance (see Fig. 1). Accordingly, specific hypotheses concerning the solution to the problems and the consequent strategies can be outlined.

The EDs in the upper left quadrant (High waiting times/Low admission rate) show a good performance with reference to the admission rates but some problems to manage the waiting times. Therefore, these EDs may primarily look at solutions inside their organization. In such circumstances managers can investigate factors related to the abovementioned third and fourth groups of potential factors affecting high ED waiting times. In particular, the list of questions (not exhaustive) that hospital managers may detect are related to the ED staff endowment, staff productivity and equipment availability. Other potential factors leading to higher waiting times can be referred to the hospital organization, such as the existence of fast track paths or the capacity of the wards to rapidly take in charge those patients who need to be hospitalized. In the case of the upper right quadrant (High waiting times/High admission rate) the EDs show problems with reference to both the admission rate and the waiting times. In these circumstances the list of questions is longer because solutions may refer not only to the ED/hospital but also to the primary care. The managers could investigate a mix of issues related to all the four groups of factors identified in literature. The problems referring to the high ED admission rate pertain to the overall organization and performance of the health care system, usually outside the ED control. In particular, the factors of the first two groups refer to i) how primary care and continuity are organized; ii) the existence and effectiveness of organizational models and clinical pathways for chronic patients. Another group of issues that may affect the situation of EDs positioned in that quadrant may concern the third and fourth groups: delays both in the admission phase (for instance, in terms of presence of fast track protocols), and in the discharge phase (due, for instance, to boarding for the admission to the wards); bottlenecks concerning for instance the imaging diagnostic services and the structural efficiency (staff productivity and staff endowment).

The EDs in the bottom right quadrant (Low waiting times/High admission rate) are efficient with reference to the waiting times, but the situation may suggest a sub-optimal resource distribution among the settings of care and a potential inappropriate answer of primary care services to the health needs of the population.

The EDs that are in the bottom left quadrant (Low waiting times/Low admission rate) are in an apparently good situation where the demand (admission rate) and the waiting times seem to be under control.

**Fig. 2:**
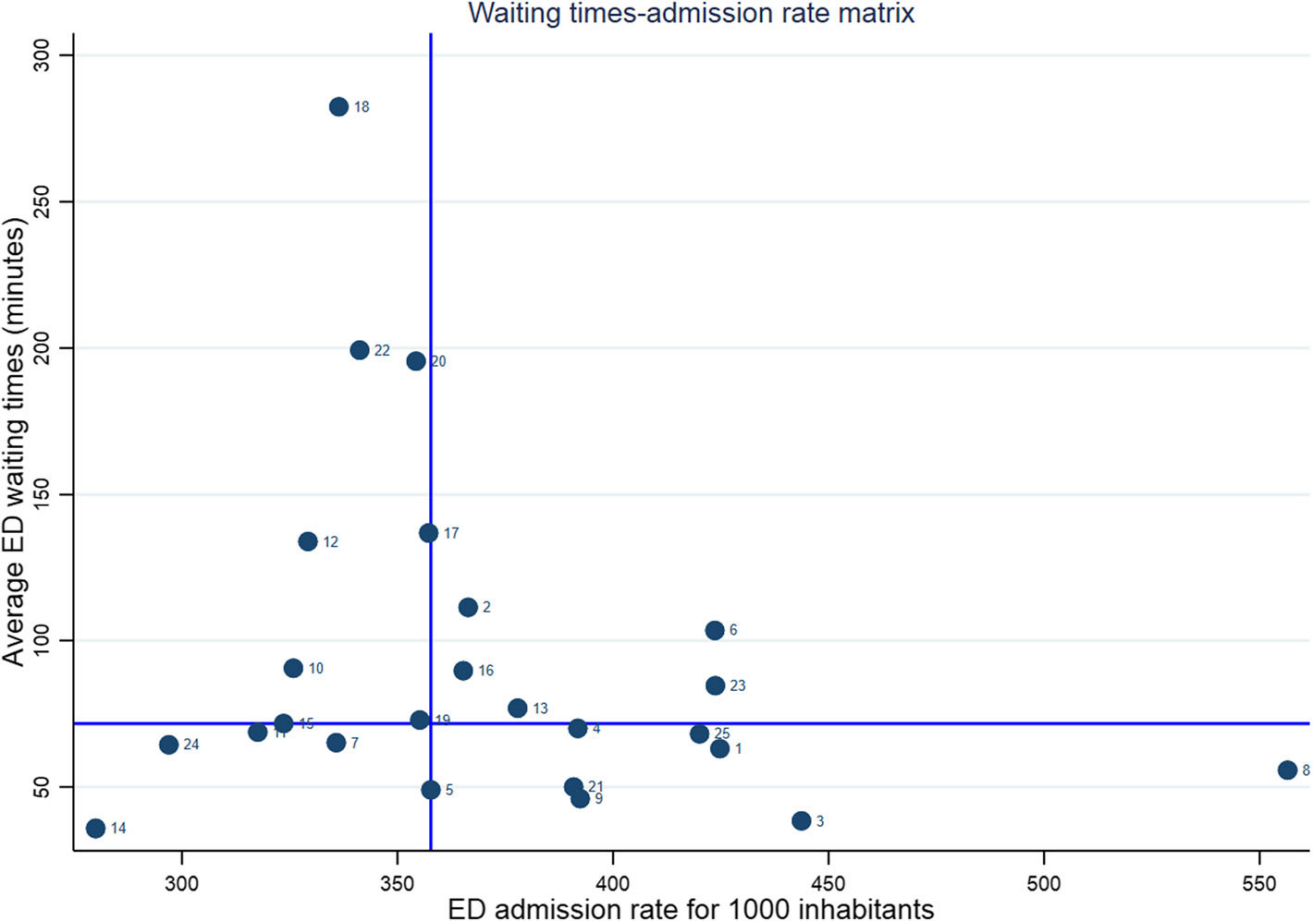
The ED waiting times-admission rate matrix for all the Tuscan EDs

Figure 2 shows how the Tuscan EDs are positioned into the matrix while Table 2 reports the descriptive statistics for each quadrant. Some distinctive traits for these four quadrants emerge from the matrix.

**Table 2:** Average characteristics of the four quadrants

***Factors***	***Quadrant upper-left***	***Quadrant upper-right***	***Quadrant bottom-left***	***Quadrant bottom-right***	***Tuscany***
*ED waiting times (minutes)*	147.93	93.26	55.06	58.56	79.32
*ED admission rate (per 1000 inhabitants)*	340.39	391.38	422.24	307.58	361.49
***Group 1*:*Continuity of care***					
*N*umber of *GPs per 1000 inhabitants*	0.75	0.61	0.70	0.71	0.66
***Group 2*:*Chronic care***					
*% of inhabitants (*≥ *16 years*) *enrolled in Tuscan chronic care programmes*	53.16	68.55	76.33	72.79	61.13
*Chronic disease management: heart failure*	168.97	147.36	129.39	145.97	151.95
*Chronic disease management: diabetes*	20.32	13.33	13.26	11.52	16.10
*Chronic disease management: obstructive pulmonary disease*	38.12	16.60	17.22	20.98	28.14
***Group 3*:*Endowment of personnel***					
*N*umber of *FTE* p*hysicians per 10*,*000 admissions*	3.83	3.41	3.91	5.06	3.98
***Group 4*:*Organization***					
*% of non-urgent treated within* four *h*ou*rs*	71.13	70.18	84.39	77.17	76.56
*% of hospitalized patients among th*os*e admitted to ED*	14.53	11.69	10.80	12.97	12.64
*% hospitalized patients within* eight *hours*	80.81	80.35	92.67	80.33	79.47
*Bed occupancy rate*	83.10	80.81	71.10	71.36	80.85
*% admissions to the observation* u*nit*	6.97	5.75	8.85	10.84	7.40
*% of hospitalized patients from the observation* u*nit*	22.56	33.07	24.28	25.74	27.75
*Observation* u*nit length of stay < 6 h*	16.69	13.83	29.53	40.67	27.78
*Observation* u*nit length of stay > 48 h*	9.00	16.75	6.92	7.68	9.88
*% of patients admitted to the ward hospitalized in the intensive care within 24* h	0.63	0.41	0.68	0.71	0.70
*% repeat admissions to the ED within 72 h*	3.04	3.08	1.90	3.67	2.93
*% patients left without being seen*	3.17	2.99	3.35	3.50	3.09

EDs belonging to the upper-left quadrant (high waiting times/low admission rate) are characterized by the highest number of General Practitioners per 1.000 inhabitants but lower performance in the chronic management indicators. It seems to suggest that the primary care is well structured in terms of number of GPs (the first group of factors) but it is not well organized to treat chronic patient (the second group of factors). Despite the lower ED admission rates EDs of this quadrant, on average it presents a number of FTE of physicians per 10,000 admissions slightly lower than the regional mean, which could be a reason behind the higher ED waiting times (the third group of factors). In addition, with reference to the organization (the fourth group of factors), these EDs show the highest percentage of hospitalized patients and also the highest bed occupancy rate; another interesting aspect that characterizes this group of EDs is the lower recourse to the observation unit. All these aspects could be among the main causes of boarding and delay in the discharge phase.

In the upper-right quadrant (high waiting times/high admission rate) EDs show a poor primary care structure, because they have the lowest rate of GPs per inhabitant (the first group of factors), but a good performance of chronic management (the second group of factors); the personnel endowment is the lowest of the four groups which can be one of the reasons for the high ED waiting times (the third group of factors); the factors related to the organization (the fourth group) show that the bed occupancy rate is on average but these EDs seem to wait more than other to hospitalize patients in the intensive care units and have less patients in observation units although for more time. These organizational reasons may lead to higher waiting times.

The bottom right quadrant (low waiting times/high admission rates) comprehends EDs with a primary care structure slightly better than the regional average and good performance of the chronic management so that the reasons of high admission rates rely on other factors. The low waiting times are coherent with an average ED endowment of personnel. The bed occupancy rate is the lowest among the four quadrants. In addition, the Observation Unit is intensively used, above all for the short stays: this could alleviate the pressure on the wards and contributing to the highest percentage of patients hospitalized within 8 h.

The bottom left quadrant (low waiting times/low admission rates) comprehends EDs characterized by an adequate number of GPs per 1.000 inhabitants, good performance on primary care related to chronic disease management, which are coherent with a low admission rate. The low waiting times may be also explained by the highest endowment of ED personnel. Moreover, the organizational factors here investigated seem useful to reduce potential problems of boarding, such as the low bed occupancy rate and the highest percentage of access to Observation unit.

### The correlation analysis

Table 3 shows the Spearman’s rank correlation matrix. It is worth to be noticed that association between ED waiting times and the ED admission rate registers a *p*-value higher than 0.10. Although, a high level of ED admission per inhabitants may lead to overcrowding, it seems that in Tuscany other factors (or a mix of them) may cloud out this relationship.

**Table 3:** Spearman’s rank correlation

	ED waiting times for inhabitants	ED admission rate	N. GPs rate	N. FTE Physicians per adm	B261	C2A2	C11A11	C11A21	C11A31	C161	C162	C163	C164	C1651A	C165	C166	C167	C168	C169	C1610	C1618	D9A
ED waiting times for inhabitants	1.00																					
ED admission rate	−0.22	1.00																				
N. GPs rate	−0.09	− 0.16	1.00																			
N. FTE Physicians per adm	−0.41*	− 0.36*	0.30	1.00																		
B261	−0.52*	0.04	−0.01	0.60*	1.00																	
C2A2	0.58*	−0.15	−0.19	− 0.33	−0.51*	1.00																
C11A11	0.60*	−0.22	0.15	−0.14	−0.37*	0.17	1.00															
C11A21	0.23	−0.01	0.30	−0.23	−0.58*	0.21	0.34	1.00														
C11A31	0.43*	−0.34	0.09	0.09	−0.52*	0.62*	0.50*	0.44*	1.00													
C161	−0.77*	−0.06	− 0.17	0.38*	0.33	−0.38*	− 0.43*	−0.21	− 0.12	1.00												
C162	−0.80*	0.14	−0.13	0.29	0.32	−0.40*	−0.60*	− 0.25	−0.36	0.79*	1.00											
C163	−0.60*	0.40*	0.23	−0.13	0.01	−0.53*	−0.34	0.21	−0.41*	0.30	0.43*	1.00										
C164	−0.32	0.18	0.40*	−0.04	0.19	−0.49*	−0.37*	0.16	−0.39*	0.03	0.03	0.56*	1.00									
C1651A	0.09	−0.24	− 0.51*	0.03	0.16	0.23	0.01	−0.10	−0.04	0.11	0.07	−0.44*	−0.43*	1.00								
C165	−0.39*	0.05	0.08	0.21	0.11	−0.00	−0.19	0.08	0.35	0.46*	0.29	0.10	0.06	−0.22	1.00							
C166	0.01	0.18	−0.06	0.12	0.02	0.28	0.13	0.00	0.17	−0.00	0.00	−0.10	− 0.48*	0.05	0.22	1.00						
C167	−0.15	−0.10	− 0.21	0.36	0.12	0.25	−0.02	−0.33	0.31	0.43*	0.34	−0.38*	−0.55*	0.10	0.44*	0.59*	1.00					
C168	−0.18	−0.12	0.43*	−0.13	− 0.34	−0.10	0.03	0.40*	0.03	0.18	−0.07	0.35	0.35	−0.31	0.05	−0.24	−0.26	1.00				
C169	0.54*	−0.46*	0.09	0.04	−0.24	0.49*	0.47*	0.43*	0.77*	−0.30	−0.44*	− 0.58*	−0.20	0.21	0.25	−0.06	0.06	−0.20	1.00			
C1610	0.01	−0.68*	−0.34	0.17	−0.02	0.42*	−0.07	− 0.17	0.31	0.30	0.07	−0.47*	−0.43*	0.63*	0.07	0.02	0.34	0.04	0.29	1.00		
C1618	−0.59*	0.15	0.1	0.34	0.28	−0.39*	−0.40*	0.16	−0.03	0.60*	0.53*	0.43*	0.30	−0.24	0.73*	0.07	0.15	0.13	−0.05	−0.12	1.00	
D9A	−0.08	0.16	0.06	−0.06	−0.11	− 0.27	0.08	0.14	−0.18	0.05	0.00	0.51*	0.24	−0.50*	0.13	0.07	−0.08	0.18	−0.32	−0.47*	0.15	1.00

**Notes:** * = *p*-value < 0.10

B261: % of inhabitants (≥ 16 years enrolled into the Tuscan chronic care programmes; C2A2: bed occupancy rate; C11A11: chronic disease management: heart failure; C11A21: chronic disease management: diabetes; C11A31: chronic disease management: obstructive pulmonary disease; % of non-urgent, non-hospitalized patients admitted to the ward within four hours; C164: % hospitalized ED patients within eight hours; C1651A: observation unit length of stay > 48 h; C165: % admissions to the observation unit; C166: % of hospitalized ED patients from the observation unit; C168: % of ED patients hospitalized in the intensive care within 24 h; C169: % of hospitalized ED patients; C1610: % repeat admissions to the ED within 72 h; C1618: observation unit length of stay < 6 h; D9A: % patients left without being seen

According to the findings of Table 3, some common patterns seem to characterize the Tuscan EDs.

With reference to the factors related to the first group, the continuity of care, investigated looking at the number of GPs per inhabitant, seems not to be related neither to the ED waiting times nor to the ED admission rate. As regards the second group of factors, most of the indicators used as proxies to analyze chronic management suggest that better performances in chronic management are also related to lower ED waiting times.

For what concerns the factors used to analyze the presence of potential bottlenecks, from one side the number of ED FTE per 10,000 admissions is negatively correlated with ED waiting times: EDs with lower FTE per 10,000 admissions show higher level of waiting times. From the other side, the number of ED FTE per 10,000 admissions shows a moderate negative association with ED admission: EDs with lower FTE per 10,000 admissions show higher level of ED admission rates. While the first association may suggest a potential resource allocation strategy at regional level, the second one suggests that when ED admission rates are high staff endowment may be not able to timely cope with the high demand.

Finally, the most represented group of factors are those related to the organization. There are several associations among variables. In particular, higher occupancy rates are associated to higher ED waiting times, this suggests that the collaboration among hospital wards and ED is an issue that Tuscan EDs have to look at. This relationship was also found in other indicators looking for similar aspects such as the prompt admission to the hospital ward. Some organizational structures such as the Observation Unit seem to help ED to cope with high waiting times. Other associations suggest that the more EDs are able to respond to non-urgent patients treated within 4 h the higher the level ED admission rates, while the higher the percentage of ED patient hospitalized, the higher are the ED waiting times (also related to the capacity of the hospital ward to board them) as well as the lower are the ED admission rates.

Correlation analysis suggests some elements that seem to characterize Tuscan EDs, however, some elements need to be further investigated on a case based. Hence, in order to help hospital and local health authority managers to disentangle which are the issues to be investigated in their situations, the ED waiting times and admission rate matrix may be used.

## Discussion

The ED is part of a service delivery system, and therefore the diagnostic logical framework presented in Fig. 1 seeks to help managers to identify the flaws and the strengths of the overall system, and the mixed strategies the local or regional health system has to apply. Indeed, the matrix allows an integrated analysis that takes into account the main factors that are bivariately associated with waiting times and admission rates, for instance the organization of EDs, ED personnel endowment and performance of primary care. The formulation of hypotheses to be investigated throughout the positioning of ED into the quadrants of the matrix and the identification of an initial list of measures already identified in literature, may support managers to address the questions that primarily can be referred to their case, thus helping them to find out the solution. Hence, this approach may support regional and hospital managers to shortlist the questions they have to answer to identify which are the potential strategies that the ED or the health system can take into account in order to better manage the waiting times. While other scholars have already highlighted the importance of some factors such as the functioning of the primary care services [17, 19–25] with a particular focus on programs related to chronic patients who are the among the ED frequent users [26, 27, 49]; the presence of bottlenecks both considering personnel [34–36] or equipment [37, 38] and other organizational aspects [37, 38], trying to find out general rules, this paper seeks to support managers and policy makers identifying those elements that specifically refers to their ED. Hence, the matrix seeks to support managers to reflect upon the application of these general rules to their case.

The descriptive statistics provide illustrative example of the suggestions coming from this diagnostic logical framework. The combined use of the matrix and the Spearman’s rank of correlation can help to understand common patterns among Tuscan EDs and more specific issues to investigate for each ED or group of EDs.

The findings of the correlation analysis highlight that resource allocation strategies and resource efficiency choices are associated to ED waiting times. In particular, FTE personnel per admission is negatively associated to ED waiting times thus suggesting that one of the factors that a regional (or meso) level of government may consider in order to better manage ED waiting times is a more equitable allocation of FTE per admissions; another aspect that may be supported at the regional level is the chronic management program, already in place in Tuscany Region. It is negatively associated to ED waiting times so that monitoring and promoting its implementation across the Tuscan districts may be one of the factors that can help managing waiting times. In addition, the regional (or meso) managers and policy makers can promote protocols to suggest the organizational choices related to hospital resource allocation (such as the use of observational units, the higher it is the lower are the ED waiting times) or to the resource efficiency (such as the bed occupancy rate, the higher it is the higher the ED waiting times) that can help containing ED waiting times.

The findings of the analysis of the four quadrants of the matrix provide empirical examples of what has been found in the Van den Heede and Van de Voorde review [50]: there is no golden rule to reduce ED waiting times or ED admission rates, so that strategies that hospital and local managers may adopt have to be personalized. Indeed, in some cases, it seems that the organizational factor that may affect the ED waiting times are the relationships with the hospital wards despite an average bed occupancy rate. While in other cases, high ED waiting times seem to be related to the primary care structure or the low performance of chronic care management.

## Conclusions

This paper adapted the waiting times-admission rate matrix already used in other services [44, 45] to the ED context also using the illustrative example of the Tuscan EDs. The matrix can work as a logical diagnostic tool to help managers to analyze the situation of their EDs.

A strength of this study is the classification of the main factors that previous researchers have identified as main determinants of ED admission rate and waiting times into a logical framework that can support managers to address the questions that primarily can be referred to their case, thus helping them to find out the solution. Indeed, the matrix may help to shortlist the issues to focus on, based on the ED positioning among the quadrants.

In addition, this diagnostic logical framework attempts to lead local and regional managers to cope with ED waiting times using a systemic approach, thus not only looking at the hospital or ED organization but considering also other factors that may affect their situation.

This study has a number of limitations. First, the analyses and results refer to the context of one Region (Tuscany) in one country (Italy). However, the logical framework proposed in this study as well as the kind of analyses conducted and the type of variables considered, may be easily replicated in other contexts, since they are derived from theory. The results may be different but the approach in detecting the situation of each group of EDs could be the same. Second, the waiting times-admission rates matrix presented in this study works well and it is a supportive source of information for policy makers only when there is the opportunity to compare performances and data of both EDs as well as primary care, continuity and hospital performances. Moreover, countries and regions may enrich their analyses including more indicators per group of factors.

Third, the matrix was presented and discussed in a workshop with the head of the EDs but it has not been used yet by policy makers and managers to detect the factors affecting ED waiting times.

Finally, it is worth highlighting that this study is purely descriptive, without any claim to derive causal inference from the analyses presented. The matrix developed, together with the correlations analysis, provide a picture of the actual situation characterizing the Tuscan EDs, and an interesting starting point to support healthcare managers and policy makers in the analyses and potentially solution to problems linked to high EDs waiting times or inappropriate admission rates.

## Data Availability

The datasets used and/or analyzed during the current study are available from the corresponding author on reasonable request.

## Abbreviations

ED: Emergency department
LHA: Local health Authority
GP: General practitioner
FTE: Full-time equivalent
B261: % Of inhabitants > = 16 years enrolled into the TUSCAN CHRONIC CARE PROGRAMS.
C2A2: Bed occupancy rate
C11A11: Chronic disease management for heart failure
C11A21: Chronic disease management for diabetes
C11A31: Chronic disease management for obstructive pulmonary disease; % of non-urgent, not hospitalized patients admitted to the ward within 4 h
C164: % Hospitalized ED patients within 8 h.
C1651A: Observation Unit length of stay > 48 h
C165: % Admissions to the Observation Unit.
C166: % Of hospitalized ED patients from the Observation Unit.
C168: % Of ED patients hospitalized in the intensive care within 24 h.
C169: % of hospitalized ED patients.
C1610: % Repeated admissions to the ED within 72 h.
C1618: Observation Unit length of stay < 6 h
D9A: % Patients left without being seen.
